# In Reply: Practicability and Diagnostic Yield of One-Stop Stroke CT with Delayed-Phase Cardiac CT in Detecting Major Cardioembolic Sources of Acute Ischemic Stroke

**DOI:** 10.1007/s00062-021-01045-x

**Published:** 2021-07-06

**Authors:** Friederike Austein, Matthias Eden, Marcus Both, Mona Salehi Ravesh, Olav Jansen, Patrick Langguth

**Affiliations:** 1 Department of Neuroradiological Intervention and Diagnostics, University Hospital Hamburg-Eppendorf, Hamburg, Germany; 2 Department of Medicine III, University of Heidelberg, Heidelberg, Germany; 3 Department for Radiology and Neuroradiology, University Hospital Schleswig-Holstein, Campus Kiel, Kiel, Germany

To the Editor.

We would like to thank Yeo et al. for sharing their valuable insights and thoughts about our work [1] in their letter to the editor [2].

The detection of cardioembolic sources of stroke (CES) can be optimized using cardiac computed tomographic (CT) imaging [3]. Some studies have already demonstrated that in adult patients with an acute cerebral infarction or transient ischemic attack, an additional cardiac CT or extended CT angiography (CTA) in the acute or early phase may provide diagnostic advantages in determining stroke etiology [4, 5]. The common aim of our work and of the studies published by different research groups is i) to increase diagnostic yield with respect to stroke etiology and ii) to improve the early decision making for the initiation of secondary prophylaxis and iii) thus to reduce the number of cryptogenic and future cerebral infarctions [6, 7].

In preparation for our study, we evaluated several published protocol variants, including non-electrocardiogram (ECG) gated cardiac CT as well as extending CTA gating up to diaphragm level according to the aforementioned literature [8]; however, using these protocols in our patients we observed more frequent limitations in terms of assessability of CES when non-ECG gated CTA was applied compared to our final protocol. In particular, there was an increased number of inappropriate images of the left atrial appendage (LAA) and other important cardiac structures, including valves, due to pulsation artifacts.

Of note, other groups used different approaches to overcome these limitations. Taina et al. described a measurement procedure of Hounsfield units to differentiate between blood stasis and thrombus within the LAA [9]. Guglielmi et al. also performed a non-gated CTA for the detection of cardioaortic sources of embolism in the acute phase of ischemic stroke. They highlighted in their conclusion that in some cases there were limitations in assessability due to suboptimal image qualities. Guglielmi et al. recommended performing further ECG gated studies to optimize image quality [10]. Hur et al. described similar results [11].

Since the number of CES was limited in the study of Yeo et al. (*n* = 3) [8] compared to our study (*n* = 10 [1]), we think that one strength of our dataset is the increased internal validity regarding the value of non-ECG gated CTA. Moreover, the delayed and ECG-triggered cardiac CT in an acute stroke setting used in our study resulted in higher diagnostic yield and image quality with potential diagnostic benefit.

Nonetheless, we agree with the suggestions of Yeo et al. that extended CTA might be sufficient in some cases; however, we are convinced considering our experience and several other studies [10, 12] that an ECG gated delayed phase cardiac CT has a superior efficiency in particular with respect to CES. In line with our observations, Apfaltrer et al. have shown that cardiac CT and transthoracic echocardiography (TTE) have a comparatively high specificity and negative predictive value for CES recurrence [13]. As TTE is not the preferred diagnostic method to detect an atrial thrombus (which is mostly detected by transesophageal echocardiography, TEE), cardiac CTA may be a valid and even superior alternative to TTE alone, since TEE has other important limitations mentioned in our manuscript [1, 5]. This advantage is further emphasized by the fact that TEE is often a limited resource and that immediate CTA is also part of standard operating procedures to identify patients suitable for endovascular treatment [14].

Beyond that there are studies that also consider anatomic variations and volumetric parameters with respect to cardiac sources of embolism, including the ones that are mentioned [15]. For example, Yaghi et al. correlated LAA morphology to stroke risk, claiming that the “chicken wing configuration” was least associated with potential embolic stroke [16].

Last but not least, we would like to point out that CES sources include causes other than atrial or ventricular thrombi, such as endocarditis, native valve diseases, and mechanical valve prothesis clotting, cardiomyopathies with remodeling and ventricular aneurysm formation, atrial myxoma, and most important, patent foramen ovale (PFO) [17]. Overall, we strongly believe that advanced imaging methods and protocols, such as dual energy CT, may help to answer some the aforementioned questions in future studies.
